# A Case of Human Immunodeficiency Virus-Positive Patient Diagnosed during the Treatment of Right Internal Iliac Pseudoaneurysm

**DOI:** 10.3400/avd.cr.20-00139

**Published:** 2021-03-25

**Authors:** Yohei Iseki, Masahiro Fujii, Dai Nishina, Shohei Mizushima, Takahiko Mine, Shin-ichiro Kumita, Yosuke Ishii, Ryuzo Bessho

**Affiliations:** 1 Department of Cardiovascular Surgery, Nippon Medical School Chiba Hokusoh Hospital, Inzai, Chiba, Japan; 2 Department of Radiology, Nippon Medical School Chiba Hokusoh Hospital, Inzai, Chiba, Japan; 3 Department of Radiology, Nippon Medical School, Tokyo, Japan; 4 Department of Cardiovascular Surgery, Nippon Medical School, Tokyo, Japan

**Keywords:** human immunodeficiency virus, internal iliac artery aneurysms, vasculitis

## Abstract

Isolated internal iliac artery aneurysms are rare, and there are no reports of human immunodeficiency virus (HIV)-related vasculitis in Japan. We report our experience with a 51-year-old man diagnosed with acquired immunodeficiency syndrome, discovered during the postoperative course when the patient exhibited remittent fever and susceptibility to infection after emergency interventional radiology therapy for a right isolated internal iliac artery aneurysm. The patient had positive treponema pallidum particle agglutination test result before admission, and tests for sexually transmitted disease showed positive results for HIV H-1 antibodies. The repeated fevers were attributed to HIV infection-related susceptibility.

## Introduction

Isolated internal iliac artery aneurysms are rare. Their etiology includes arteriosclerosis, collagen disease, hereditary factors, connective tissue disease, trauma, and infection. Meanwhile, despite the increasing number of patients with human immunodeficiency virus (HIV) infections and acquired immunodeficiency syndrome (AIDS) in Japan, we could find any report of HIV-related vasculitis. In this study, we report our experience with a patient diagnosed with AIDS due to postoperative susceptibility to infection and exhibited what we believe was a right isolated internal iliac artery aneurysm caused by HIV-related vasculitis, along with a discussion of the literature. Written informed consent was obtained from the patient before preparation of the case report.

## Case Report

The patient was a 51-year-old man who had visited a local doctor for shortness of breath 3 weeks prior and was referred to our hospital for possible anemia. Two days before presentation, he experienced lower-right abdominal pain and sought emergency outpatient care when it worsened. He had a history of meningitis when he was 40 years old and had a family history of chronic aortic dissection on the maternal side.

Physical findings upon arrival at the hospital were as follows: body temperature, 39.0°C; blood pressure, 120/80 mmHg; pulse rate, 95/min; and oxygen saturation, 98% (room air) and with spontaneous pain and tenderness at the lower-right abdomen.

The results of the blood test were as follows: white blood cell count (WBC), 6700/µL; red blood cell count, 300×10^4^/µL; hemoglobin, 9.1 g/dL; hematocrit, 27.4%; platelets, 7.5×10^4^/µL; prothrombin time/international normalized ratio, 1.20; activated partial thromboplastin time, 49.7 s; D-dimer, 2.2 µg/mL; aspartate aminotransferase, 33 U/L; alanine aminotransferase, 14 U/L; lactate dehydrogenase, 437 U/L; creatine kinase, 123 U/L; total protein, 8.1 g/dL; albumin, 3.2 g/dL; blood urea nitrogen, 19.7 mg/dL; creatinine, 1.45 mg/dL; sodium, 135 mEq/L; potassium, 3.6 mEq/L; chlorine, 102 mEq/L; C-reactive protein, 5.55 mg/dL; and procalcitonin, 1.52 ng/mL.

**Figure 1:**
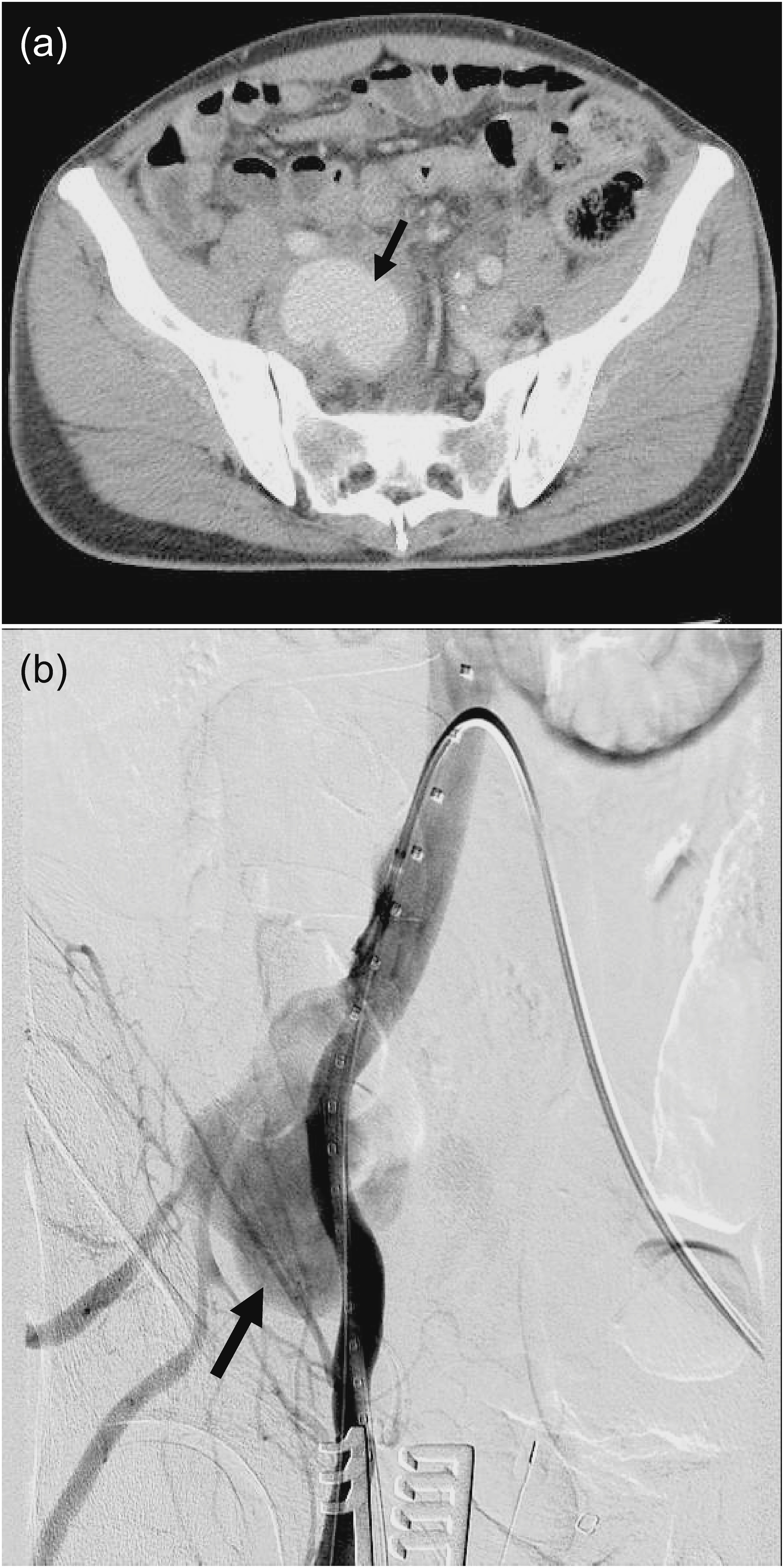
Fig. 1 (**A**) Abdominal contrast-enhanced computed tomography showing a pseudoaneurysm (60×45 mm) in the right internal iliac artery (arrow). (**B**) Contrast imaging of the right common iliac artery (pretreatment) showing a pseudoaneurysm in the right internal iliac artery (arrow), similar to the abdominal computed tomography finding.

Chest X-ray did not show any significant findings, but abdominal X-ray revealed a niveau formation. Contrast-enhanced abdominal computed tomography (CT) showed a pseudoaneurysm (6×4.5 cm) near the bifurcation of the right internal iliac artery, with increased intensity of the surrounding adipose tissue (**Fig. 1A**). Contrast imaging of the right common iliac artery (pretreatment) showed a pseudoaneurysm in the right internal iliac artery, which is similar to the abdominal CT finding (**Fig. 1B**). Right pyelocaliectasis was also observed.

**Figure 2:**
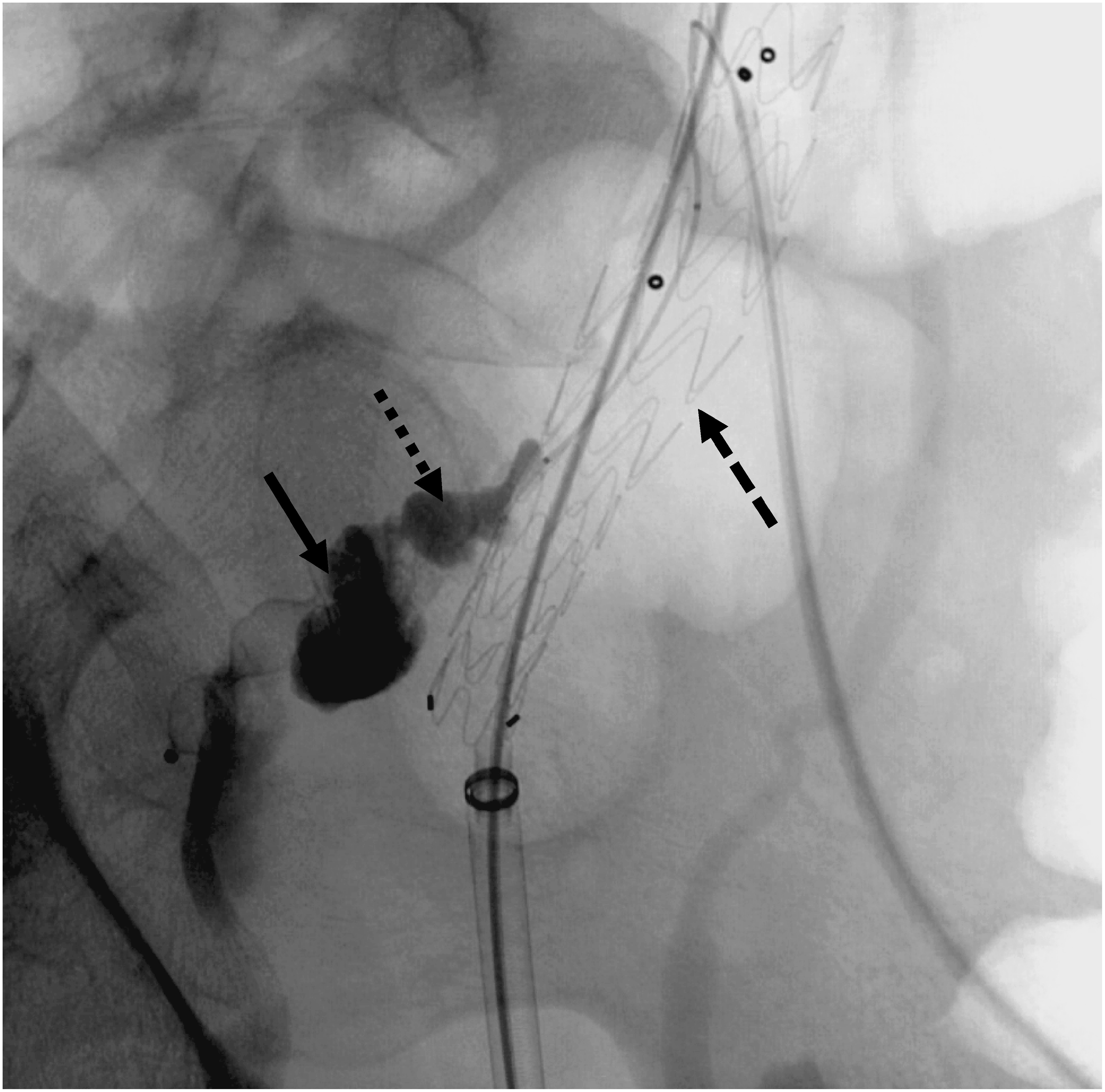
Fig. 2 Contrast imaging of the right common iliac artery (posttreatment).

Emergency interventional radiology (IVR) was performed for the pseudoaneurysm of the right internal iliac artery. The left femoral artery was punctured, contrast enhancement of the right common iliac artery was performed, and an AMPLATZER Vascular Plug II (10 mm) (AGA Medical, Golden Valley, MN, USA) was inserted into the peripheral side of the right internal iliac artery for embolization. An Endurant II (16–10 mm) (Medtronic Cardiovascular, Santa Rosa, CA, USA) was then placed from the right common iliac artery to the right external iliac artery. Additional embolization was performed by injecting a mixture of *n*-butyl-2-cyanoacrylate and lipiodol (3 mL) (Guerbet-Japan, Tokyo, Japan) into the pseudoaneurysm. The procedure was finished after confirming that the contrast effect in the aneurysm had disappeared (**Fig. 2**).

Solid arrow, AMPLATZER Vascular Plug II; broken-line arrow, Endurant II; dotted-line arrow, *n*-butyl-2-cyanoacrylate, and lipiodol mixture

**Figure 3:**
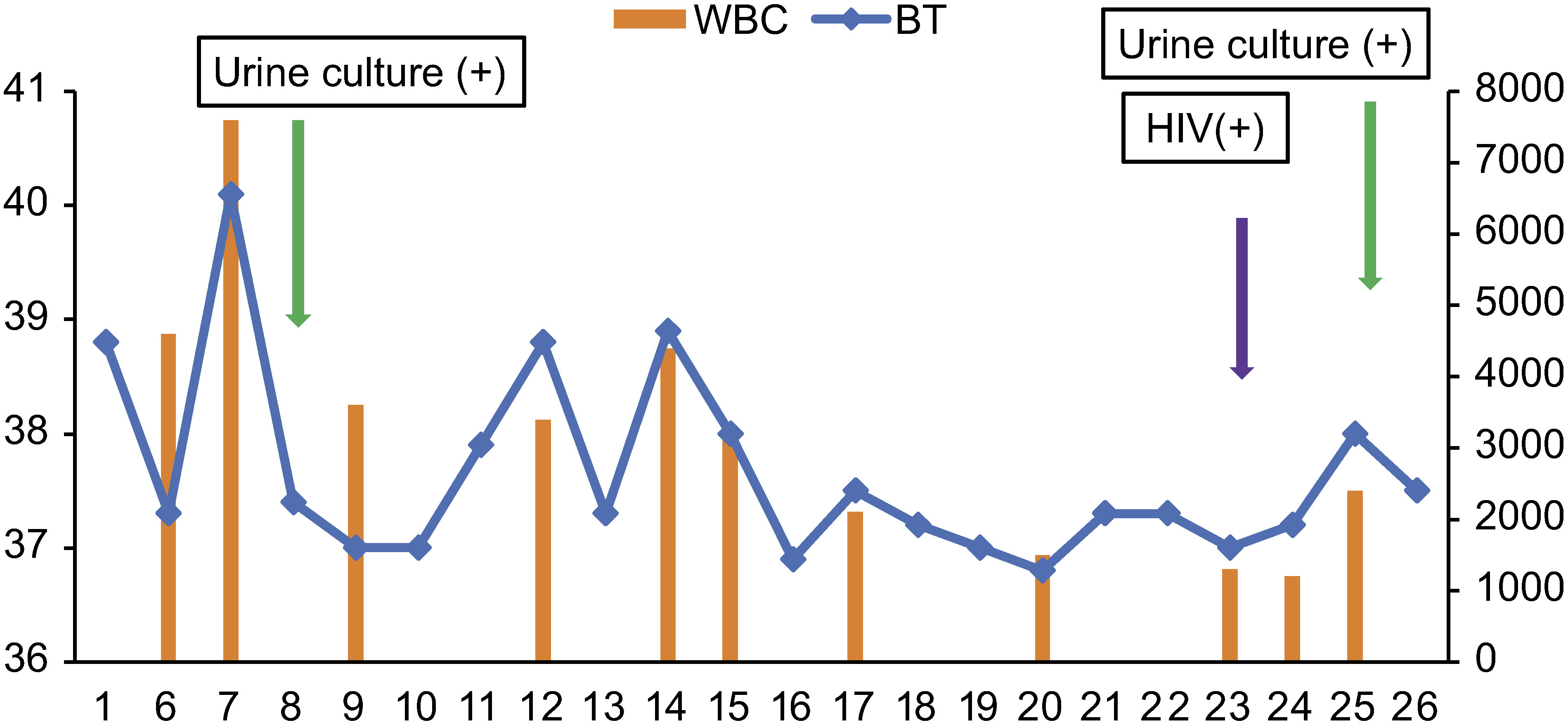
Fig. 3 Postoperative course. The horizontal axis shows hospital days, the left vertical axis shows body temperature (°C), and the right vertical axis shows the WBC per microliter. WBC, white blood cell count; BT, body temperature

After surgery, the patient exhibited a delayed inflammatory response with remittent fever and pancytopenia. Escherichia coli was detected in a urine culture, and since dilatation of the renal calyx was observed on preoperative abdominal CT, we diagnosed a urinary tract infection caused by ureteral pressure and blockage from the pseudoaneurysm. The infection was treated with a sensitive antimicrobial agent. Blood flow was not observed on postoperative CT, and since the pseudoaneurysm did not considerably shrink, a renal fistula was created. After the renal fistula was created, the patient’s urinalysis results improved; however, the patient experienced repeated bouts of fever. During this time, the WBC remained below the normal range (**Fig. 3**). Since the patient had a positive Treponema pallidum latex agglutination result before admission, further examinations for sexually transmitted disease were performed, which showed that he was positive for HIV H-1 antibodies, with reduced CD4 level (<45/µL) and a CD4/CD8 ratio of 0.3. The repeated fevers were thought to be due to susceptibility to infection subsequent to the HIV infection. Despite the absence of any indicator diseases, the low WBC raised concerns about opportunistic infections and AIDS-defining cancers. On Day 26 after surgery, he was transferred to a hospital designated for infectious diseases for specialist treatment.

## Discussion

Solitary iliac artery aneurysm without abdominal aortic aneurysm is rare, representing less than 1% of aneurysms. Surgical mortality in ruptured cases is reported to be 20% to 57%.^1)^ Cases are often asymptomatic until rupture occurs, although possible symptoms include abdominal, buttock, and inguinal pain; frequent urination, difficult urination, and hydronephrosis due to pressure on the ureter or bladder; and difficulty defecating, intestinal obstruction, and blood in the stool due to pressure on the rectum or sigmoid colon.

Although most internal iliac aneurysms are due to atherosclerosis, other causes include collagen disease, hereditary connective tissue disease, trauma, and infection. In this case, there were no preceding infectious symptoms and both the blood and urine cultures were negative on admission. HIV infection was discovered during the course of treatment, and the associated vasculitis associated was believed to be the cause of the rupture and formation of a pseudoaneurysm. Unfortunately, pathological specimens were not available for determination of characteristic arterial features.

HIV-related vasculitis is a rare complication reported to occur in approximately 1% of all cases. It is more common in immunosuppressed states when the CD4 T lymphocyte count is <200/mL.^2)^

The onset mechanism of HIV-related vasculitis is thought to involve not only direct invasion of blood vessels or immunological elements but also (1) pathogen-associated molecular pattern molecules produced from intestinal microorganisms that act on immune cells to create a chronic inflammatory state that causes endothelial damage and (2) oxidative stress associated with an infection that produces oxidized low-density lipoprotein that acts on immune cells to produce endothelial damage and chronic inflammation, and the combination of the above is thought to cause intravascular atherosclerosis.^3)^ The inflammation in HIV-related vasculitis mainly occurs in the tunica adventitia, which causes aneurysms and vascular blockage.^4)^ In addition, the incidence rate has been found to increase when highly active antiretroviral therapy for AIDS is suspended.

The aneurysms and arterial blockage caused by HIV-related vasculitis are usually found in peripheral arteries, often the carotid or superficial femoral artery.^4,5)^ In addition, aneurysms are often found in multiple sites or in sites rarely seen in atherosclerotic aneurysms.^6)^ While IVR treatment for ruptured aneurysms in HIV-infected patients remains under debate, there are very few reports regarding treatment of HIV patients with large vessel aneurysms. This patient was diagnosed with idiopathic pseudoaneurysm because he had no prior infection, had a negative blood culture, and was not considered HIV-infected. Therefore, we selected IVR therapy. Fortunately, our experience showed that IVR treatment in such a patient can be safely performed without any complications. Outpatient follow-up assessments have been performed every 3 months, and the outcome at two and a half years postoperatively is good.

There have been a few reports of HIV-related vasculitis as a complication of HIV infection, mainly from African countries^2–8)^; we could not find any case reports from Japan. However, the Japanese Ministry of Health, Labour and Welfare statistics shows an increasing trend in the number of new HIV infections and AIDS patients, which is similar to the increasing trend across East Asia. Thus, the number of HIV-related vasculitis cases in Japan is expected to increase.

In this case, HIV infection was discovered during the postoperative course when the patient exhibited susceptibility to infection after emergency IVR therapy for a pseudoaneurysm. Fortunately, the patient was referred for specialist treatment before the onset of opportunistic infections. In cases of isolated or multiple peripheral aneurysms or arterial ruptures when HIV infection is suspected due to the patient’s background, or if the patient has HIV, regular imaging examinations to search for vasculitis symptoms should be considered.

## Conclusion

We experienced a case of pseudoaneurysm of the right internal iliac artery caused by HIV-related vasculitis in a patient who was found infected during the postoperative course. With the increasing number of HIV/AIDS patients, the possibility of HIV infection needs to be considered when treating patients with lower-limb region or juvenile aneurysms.
